# Spectrum of Mutations in *NDP* Resulting in Ocular Disease; a Systematic Review

**DOI:** 10.3389/fgene.2022.884722

**Published:** 2022-05-16

**Authors:** James Wawrzynski, Aara Patel, Abdul Badran, Isaac Dowell, Robert Henderson, Jane C. Sowden

**Affiliations:** ^1^ UCL Great Ormond Street Institute of Child Health, National Institute for Health and Care Research, University College London, London, United Kingdom; ^2^ Great Ormond Street Hospital Biomedical Research Centre, London, United Kingdom; ^3^ Moorfields Eye Hospital, London, United Kingdom; ^4^ University of Bristol, London, United Kingdom

**Keywords:** norrie disease, FEVR, retinopathy of prematurity, coats disease, NDP, retinal detachment (RD), hearing loss, persistent foetal vasculature

## Abstract

**Aims and Rationale:** The inner retina is supplied by three intraretinal capillary plexi whereas the outer retina is supplied by the choroidal circulation: NDP is essential for normal intraretinal vascularisation. Pathogenic variants in NDP (Xp11.3) may result in either a severe retinal phenotype associated with hearing loss (Norrie Disease) or a moderate retinal phenotype (Familial Exudative Vitreoretinopathy, FEVR). However, little is known about whether the nature or location of the NDP variant is predictive of severity. In this systematic review we summarise all reported NDP variants and draw conclusions about whether the nature of the NDP variant is predictive of the severity of the resulting ocular pathology and associated hearing loss and intellectual disability.

**Findings:** 201 different variants in the NDP gene have been reported as disease-causing. The pathological phenotype that may result from a disease-causing NDP variant is quite diverse but generally comprises a consistent cluster of features (retinal hypovascularisation, exudation, persistent foetal vasculature, tractional/exudative retinal detachment, intellectual disability and hearing loss) that vary predictably with severity. Previous reviews have found no clear pattern in the nature of NDP mutations that cause either FEVR or Norrie disease, with the exception that mutations affecting cysteine residues have been associated with Norrie Disease and that visual loss amongst patients with Norrie disease tends to be more severe if the NDP mutation results in an early termination of translation as opposed to a missense related amino acid change. A key limitation of previous reviews has been variability in the case definition of Norrie disease and FEVR amongst authors. We thus reclassified patients into two groups based only on the severity of their retinal disease. Of the reported pathogenic variants that have been described in more than one patient, we found that any given variant caused an equivalent severity of retinopathy each time it was reported with very few exceptions. We therefore conclude that specific NDP mutations generally result in a consistent retinal phenotype each time they arise. Reports by different authors of the same variant causing either FEVR or Norrie disease conflict primarily due to variability in the authors’ respective case definitions rather than true differences in disease severity.

## 1 Introduction

The retina is a multi-layered light-sensitive tissue lining the back of the eye. Its function is to detect light focused by the cornea and lens, transduce it into a neuronal signal, perform basic image processing and then to convey the information as electrical activity through the optic nerve to the brain. The retina is anatomically organised into inner and outer parts. The outer retina is comprised of photoreceptors (responsible for phototransduction) whereas the inner retina is comprised of bipolar cells, horizontal cells and amacrine cells (responsible for image processing) as well as ganglion cell bodies (whose axons make up the optic nerve) and Müller cells (responsible for metabolic support and homeostasis).

### 1.1 Normal Retinal Vascularisation

In humans, the inner retina is supplied by three intraretinal capillary plexi that originate from the central retinal artery whereas the outer retina is supplied by the choroidal circulation. A well developed and functioning inner retinal blood supply is essential for normal retinal function (Ye et al., 2009).

During development of the eye in the human foetus, the choroidal circulation develops to supply the outer retina whereas the inner retina and lens are initially supplied by the hyaloid vasculature. Retinal angiogenesis then occurs between the 15th and 40th week of gestation resulting in the formation of three intraretinal capillary plexi to supply the inner retina. The retinal circulation ultimately takes over the function of the hyaloid vasculature, leading to its regression before birth.

Retinal angiogenesis in humans begins with sprouting at the optic disc in the 15th week of gestation, followed by radial extension of the superficial vasculature towards the retinal periphery. At week 25, downgrowth from the superficial vascular network begins to form the intermediate and deep capillary layers, also spreading in a radial fashion. By the 36th to 40th week of gestation, retinal angiogenesis is typically complete (Lutty and McLeod, 2018). In this review we summarise the impact of reported variants in the NDP gene on this process and on the ocular pathology sequelae.

One of the most important factors driving retinal angiogenesis is the norrin/FZD4 signalling pathway, which has been well characterised and resembles the canonical WNT signalling pathway (MacDonald et al., 2009): norrin, a 133 amino acid cystine knot growth factor, is secreted by the Müller glia and activates its target; FZD4 receptors on vascular endothelial tip cells. FZD4 activation (by dimerisation) acts in concert with co-receptors LRP5, LRP6 and TSPAN12 to recruit the scaffolding protein Dishevelled (DVL) resulting in the removal of Axin1 (AXIN1) from the beta catenin phosphorylation/destruction complex, therefore ubiquitination of beta catenin ceases and cytosolic levels of beta catenin rise. Beta catenin enters the nucleus, where it interacts with the T-cell factor (TCF)/lymphoid enhancing factor (LEF) family of transcription factors and is thus able to promote the transcription of norrin responsive genes that orchestrate normal retinal vascular development (MacDonald et al., 2009). Expression of Ndp is increased by low oxygen tension (Ding et al., 2008; Ohlmann et al., 2010; Tokunaga et al., 2013) and in the biallelic Lrp5 knockout mouse (Chen et al., 2012). Ndp expression is also under the control of Hedgehog signalling in retinal progenitor cells, which acts as a necessary/permissive factor. However the role of Hedgehog signalling in normal/physiological development is uncertain (McNeill et al., 2013).

### 1.2 Pathogenic Variants in Several Genes Cause Incomplete Retinal Vascularisation

In patients with poorly developed retinal vasculature that have a genetic diagnosis, the most commonly identified cause is a pathogenic variant in a component of the norrin signalling pathway (NDP, FZD4, TSPAN 12, LRP5, LRP6 or CTNNB1) (Wen et al., 2015). Biallelic or hemizygous mutations in NDP (Xp11.3) often result in a severe phenotype (Wu et al., 2007), suggesting little or no redundancy within the signalling cascade at the intercellular level. In addition, pathogenic variants have been identified in a handful of other genes (ZNF408, KIF11, JAG1, RCBTB1 and ATOH7(Xiao et al., 2019; Zhang et al., 2020)), which are involved in various pathways independent of NDP signalling but also necessary for retinal angiogenesis. Mutations in these have been found to result in a similar phenotype. For example, Kif11 is a spindle motor protein that contributes to the assembly of the bipolar spindle during mitosis and is therefore important in cell division, which is in turn essential for angiogenesis (Sawin et al., 1992).

### 1.3 The Retinal Phenotype Resulting From Pathogenic Variants in NDP

Animal models demonstrate that in the absence of norrin, radial extension of the superficial retinal vasculature is significantly slowed and the vessels have a porous blood-retinal barrier. In addition, the intermediate and deep retinal capillary layers fail to develop. In the mouse, this results in an electronegative ERG (Luhmann et al., 2005; Schäfer et al., 2009).

In human patients with NDP pathogenic variants, the poorly vascularised retina becomes hypoxic and begins to secrete vascular endothelial growth factor (VEGF) (Rattner et al., 2014). In severe cases, this results in retinal neovascularisation followed inevitably by bilateral mixed tractional and exudative retinal detachment. Persistence of the foetal vasculature is common (Apple et al., 1974). Such severe cases are often associated with hearing loss in the second or third decade of life and with intellectual disability in 30% of cases (Halpin et al., 2005; Smith et al., 2012). Peripheral vascular disease, epilepsy and growth retardation have also been reported (Chen et al., 1993; Smith et al., 2012). In less severe cases VEGF expression, combined with reduced norrin signalling results in a porous blood-retinal barrier and subretinal exudation. In the mildest cases, asymptomatic peripheral non-perfusion on fluoresceine angiography may be the only examination finding (Shukla et al., 2003).

Classically, patients with bilateral tractional/exudative detachments at presentation in infancy have been diagnosed with Norrie Disease (ND), whereas patients with milder phenotypes ranging from peripheral non-perfusion and telangiectasia to unilateral exudative detachment with less severe disease in the contralateral eye have been diagnosed with familial exudative vitreoretinopathy (FEVR), grade 1 to 5 (Pendergast and Trese, 1998), Table 1. These are both terms that predate modern genetic medicine (Norrie, 1927; Warburg, 1961; Criswick and Schepens, 1969) and we hypothesise that in fact a continuum exists between the two conditions, with severity at least in part dependant on the nature of the causative NDP variant.

**TABLE 1:** Classification of FEVR adapted from Pendergast and Trese, 1998.

Stage	Clinical Features
1	Avascular retinal periphery without extraretinal vascularization
2	Avascular retinal periphery with extraretinal vascularization
a	Without exudate
b	With exudate
3	Retinal detachment; subtotal not involving he fovea
a	Primarily exudative
b	Primarily tractional
4	Retinal detachment; subtotal involving the fovea
a	Primarily exudative
b	Primarily tractional
5	Retinal detachment; total
a	Open funnel
b	Closed funnel

Since Berger first reported NDP as the causative gene in Norrie Disease (Berger et al., 1992), many variants in NDP have been reported over the past 3 decades in patients diagnosed with both ND and FEVR as well as Coats disease and retinopathy of prematurity (ROP). Standard Orphanet (orpha.net) definitions for these conditions are provided in Table 2. Norrie disease, FEVR, ROP and Coats disease are all thought to share a similar pathophysiological cause; there is inadequate activation of the norrin- > FZD4, LRP5/6, TSPAN12 signalling pathway resulting in insufficient beta catenin stabilisation and therefore abnormal expression of downstream genes responsible for normal retinal vascularisation and the maintenance of the blood retinal barrier (BRB) (MacDonald et al., 2009). Insufficient retinal vascularisation initiates a cascade of secondary events driven by hypoxia, increased VEGF secretion, resulting in aberrant neovascularisation, pre-retinal vitreous fibrosis, recurrent vitreous haemorrhage and tractional retinal detachment (Pendergast and Trese, 1998; Ash et al., 2005). In addition, VEGF secretion unopposed by norrin signalling results in a loss of integrity of the BRB leading to subretinal exudation and exudative retinal detachment (Rattner et al., 2014; Díaz-Coránguez et al., 2020). Other secondary effects of advanced disease include microphthalmia, cataract, nystagmus and corneal opacity. The primary and secondary clinical features of the above conditions (as defined by orphanet) are reflected in the HPO terms associated with NDP (Table 3) (Köhler et al., 2021).

**TABLE 2:** Definitions of the key conditions that can arise as a result of an NDP mutation.

Disease	Definition	ICD-10 Code	OMIM Reference
Norrie Disease	A rare developmental defect during embryogenesis characterized by abnormal retinal development with congenital blindness. Common associated manifestations include sensorineural hearing loss and developmental delay, intellectual disability and/or behavioral disorders	H35.5	310,600
Familial Exudative Vitreoretinopathy	Familial exudative vitreoretinopathy (FEVR) is a rare hereditary vitreoretinal disorder characterized by abnormal or incomplete vascularization of the peripheral retina leading to variable clinical manifestations ranging from no effects to minor anomalies, or even retinal detachment with blindness	H35.0	305,390
Coats disease	Coats disease (CD) is an idiopathic disorder characterized by retinal telangiectasia with deposition of intraretinal or subretinal exudates, potentially leading to retinal detachment and unilateral blindness. CD is classically an isolated and unilateral condition affecting otherwise healthy young children	H35.0	300,216
Retinopathy of Prematurity	A rare retinal vasoproliferative disease affecting preterm infants characterized initially by a delay in physiologic retinal vascular development and compromised physiologic vascularity, and subsequently by aberrant angiogenesis in the form of intravitreal neovascularization	H35.1	n/a

**TABLE 3:** Human Phenotype Ontology (HPO) terms associated with mutations in the NDP gene.

HPO Term ID	HPO Term Name
HP:0000717	Autism
HP:0002169	Clonus
HP:0007710	Peripheral vitreous opacities
HP:0007902	Vitreous hemorrhage
HP:0007663	Reduced visual acuity
HP:0000028	Cryptorchidism
HP:0008046	Abnormal retinal vascular morphology
HP:0000718	Aggressive behavior
HP:0000541	Retinal detachment
HP:0000233	Thin vermilion border
HP:0040049	Macular edema
HP:0000252	Microcephaly
HP:0000593	Abnormal anterior chamber morphology
HP:0001508	Failure to thrive
HP:0000823	Delayed puberty
HP:0000709	Psychosis
HP:0000733	Stereotypy
HP:0000533	Chorioretinal atrophy
HP:0007676	Hypoplasia of the iris
HP:0007973	Retinal dysplasia
HP:0001083	Ectopia lentis
HP:0002353	EEG abnormality
HP:0008063	Aplasia/Hypoplasia of the lens
HP:0000407	Sensorineural hearing impairment
HP:0001270	Motor delay
HP:0001493	Falciform retinal fold
HP:0002120	Cerebral cortical atrophy
HP:0001136	Retinal arteriolar tortuosity
HP:0004349	Reduced bone mineral density
HP:0012230	Rhegmatogenous retinal detachment
HP:0011039	Abnormality of the helix
HP:0006887	Intellectual disability, progressive
HP:0005293	Venous insufficiency
HP:0100718	Uterine rupture
HP:0000647	Sclerocornea
HP:0000490	Deeply set eye
HP:0004327	Abnormal vitreous humor morphology
HP:0000532	Abnormal chorioretinal morphology
HP:0000648	Optic atrophy
HP:0007018	Attention deficit hyperactivity disorder
HP:0001324	Muscle weakness
HP:0001518	Small for gestational age
HP:0000375	Abnormal cochlea morphology
HP:0000708	Behavioral abnormality
HP:0002076	Migraine
HP:0004326	Cachexia
HP:0001276	Hypertonia
HP:0007685	Peripheral retinal avascularization
HP:0030496	Macular exudate
HP:0000501	Glaucoma
HP:0100014	Epiretinal membrane
HP:0030503	Macular telangiectasia
HP:0000615	Abnormal pupil morphology
HP:0001419	X-linked recessive inheritance
HP:0001250	Seizure
HP:0001622	Premature birth
HP:0011530	Retinal hole
HP:0010662	Abnormality of the diencephalon
HP:0012841	Retinal vascular tortuosity
HP:0000365	Hearing impairment
HP:0000739	Anxiety
HP:0031526	Subretinal fluid
HP:0100742	Vascular neoplasm
HP:0007833	Anterior chamber synechiae
HP:0007968	Remnants of the hyaloid vascular system
HP:0100012	Neoplasm of the eye
HP:0007360	Aplasia/Hypoplasia of the cerebellum
HP:0100639	Erectile dysfunction
HP:0000446	Narrow nasal bridge
HP:0000738	Hallucinations
HP:0000639	Nystagmus
HP:0002360	Sleep disturbance
HP:0000518	Cataract
HP:0002650	Scoliosis
HP:0000737	Irritability
HP:0000411	Protruding ear
HP:0012795	Abnormality of the optic disc
HP:0001252	Hypotonia
HP:0000594	Shallow anterior chamber
HP:0030666	Retinal neovascularization
HP:0000726	Dementia
HP:0100832	Vitreous floaters
HP:0001103	Abnormal macular morphology
HP:0011532	Subretinal exudate
HP:0100716	Self-injurious behavior
HP:0008053	Aplasia/Hypoplasia of the iris
HP:0001004	Lymphedema
HP:0001141	Severely reduced visual acuity
HP:0030490	Exudative vitreoretinopathy
HP:0000601	Hypotelorism
HP:0007773	Vitreoretinopathy
HP:0010978	Abnormality of immune system physiology
HP:0011342	Mild global developmental delay
HP:0007917	Tractional retinal detachment
HP:0008052	Retinal fold
HP:0000486	Strabismus
HP:0002376	Developmental regression
HP:0007957	Corneal opacity
HP:0007759	Opacification of the corneal stroma
HP:0000568	Microphthalmia
HP:0000400	Macrotia
HP:0000819	Diabetes mellitus
HP:0000618	Blindness
HP:0007989	Intraretinal exudate
HP:0001256	Intellectual disability, mild
HP:0000272	Malar flattening
HP:0001347	Hyperreflexia

The cause of inadequate norrin signalling in FEVR and Norrie disease is a primary deficiency due to a genetic mutation in the norrin- > FZD4, LRP5/6, TSPAN12 signalling pathway, affecting retinal vascular development beginning *in utero*. In ROP, development is thought to be normal until premature birth occurs, causing an oxygen-induced suppression of norrin secretion and hence arresting vascular growth (Tokunaga et al., 2013; Hartnett, 2015; Madaan et al., 2019) leading to subsequent retinal hypoxia. There is therefore likely to be an interaction between the environment (hyperoxia) and the patients’ genotype (variants in the norrin signalling pathway) that may affect the likelihood or severity of ROP. Coats disease is unilateral (by definition) and therefore the aetiology of the reduction in activity of the norrin signalling pathway may be driven by a combination of germline and somatic genetic changes (Black et al., 1999).

This review summarises all variants in NDP that have been associated with ocular pathology to date including ND, FEVR, Coats disease and ROP and examines the correlation between the amino acid change and the severity of the clinical phenotype. We aim to determine if the amino acid change and its effect on norrin protein structure is predictive of the resulting disease (FEVR or Norrie Disease). In particular we classify patients into “severe” and “moderate” retinopathy (rather than Norrie disease and FEVR) based upon objective clinical examination findings as reported in each case report in the literature in order to standardise reporting across all authors.

## 2 Methods

A systematic search of PubMed was conducted in order to review the clinical phenotypes associated with all pathogenic variants in NDP reported until first May 2020. Search terms used were “NDP mutation” OR “NDP variant” OR “Norrie disease pseudoglioma mutation” OR “Norrie disease pseudoglioma variant”. Titles were then screened to rule out papers unrelated to variants in the NDP gene. All remaining papers were included in the review. In addition, further papers were included where relevant if they were referenced by NDP et al., 2021 https://databases.lovd.nl/shared/genes/NDP, https://databases.lovd.nl/shared/transcripts/NDP.

Each reported mutation in NDP was tabulated, along with its corresponding amino acid change if the mutation was in the coding region. Consistent nomenclature of coding DNA mutation consequences was ensured through the use of mutalyser (Lefter et al., 2021) using the NCBI reference sequence and the search term “NG_009,832.1(NDP):c.___” followed by the cDNA change. A common variation in nomenclature was for authors to number nucleic acid changes from the beginning of exon one rather than from the beginning of the coding sequence. Each missense variant underwent in-silico pathogenicity prediction using polyphen, SIFT and CADD scoring. The clinical phenotype reported in each case was also recorded in as much detail as was available according to the following headings: Reported clinical diagnosis [Norrie disease, FEVR, Coats Disease, retinopathy of prematurity, other]; Retinal phenotype [Avascular periphery or anomalous intraretinal vascularization, Avascular periphery or anomalous intraretinal vascularization with exudate or leakage, Avascular periphery with vitreoretinal neovascularisation, Avascular periphery with vitreoretinal neovascularisation and exudation, Fovea-on retinal detachment, Fovea-off retinal detachment, Total retinal detachment, Phthisis bulbi]; Presence or absence of hearing loss [present, absent (patient <30 years old), absent (patient >30 years old)]; Presence of intellectual disability [present, absent]; Presence of any other syndromic features. We used Human Phenotype Ontology (HPO) as the standardised phenotype vocabulary for recording patient phenotypes.

**FIGURE 1:**
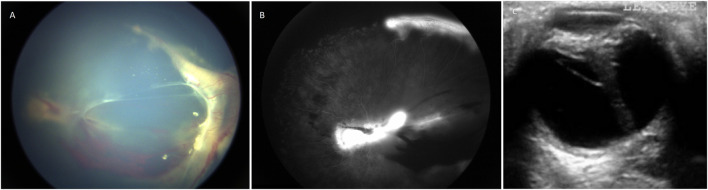
**(A)** Colour fundus photograph showing an example of moderate retinopathy. **(B)** Fluoresceine angiogram of the same eye. **(C)** An ultrasound B scan showing an example of severe retinopathy. This child with Norrie disease has a total retinal detachment in a closed funnel configuration.

In order to remove the effect of variable nosology with respect to whether a patients’ retinal condition ought to be labelled ‘Norrie disease’ or ‘FEVR’ by hundreds of authors from many countries around the world over the past 20 years, we devised a new classification system. Patients were classified as “severe” if at presentation they had bilateral total retinal detachments, if one eye was phthisical and the other had a total retinal detachment or if both eyes were phthisical. Otherwise, patients with at least one eye with a sub-total retinal detachment or more mild disease than that were classified as “moderate”. Examples are shown in Figure 1. This classification was chosen as it enabled consistency in our analysis whilst respecting the original diagnostic label in the greatest possible number of cases.

These criteria for classification of patients’ retinal disease resulted in the greatest number of patients originally diagnosed with Norrie disease being classified as ‘severe’ as well as maximising the number of patients originally diagnosed with FEVR patients being classified as ‘moderate’, hence making our classification as consistent as possible with previous papers. 38% were reclassified using these criteria. If the severity of the retinal phenotype could not be adequately determined from the paper, these patients were excluded from the analysis.

Common/likely benign single nucleotide polymorphisms were identified using the Genome Aggregation Database, gnomAD (Karczewski et al., 2020) browser in order to present a complete picture of the effect of all reported variants in NDP on the phenotype. Polymorphisms that are likely to be non-pathogenic were identified as such if either the cDNA change was synonymous or if a missense mutation was found to be “benign” and “tolerated” based on results of the Polyphen and SIFT pathogenicity predictors respectively (Adzhubei et al., 2010; Sim et al., 2012) and if they were not predicted to affect splicing using the program Splice AI (Jaganathan et al., 2019).

## 3 Results

The systematic search of Pubmed found 447 papers. Of these papers and their references, 106 described case reports of patients with NDP mutations associated with retinal disease. Within these papers, the authors reported 201 unique NDP variants associated with retinal disease. All caused vitreoretinal pathology characteristic of incomplete retinal vascular development and a poorly functioning blood-retinal barrier. 58 (29%) of these pathogenic variants have been reported in multiple studies, however the majority have been reported once or in only a single family.

According to the original authors’ diagnoses, NDP variants were associated with Norrie Disease (140 variants), FEVR (62 variants), ROP (16 variants), Coats disease (3 variants) and bilateral Persistent Foetal Vasculature Syndrome (1 variant). Sixteen variants were associated with both FEVR and Norrie Disease, all three variants associated with Coats were also associated with either Norrie disease or severe cases of FEVR. One variant was associated with both ROP and FEVR. 71 NDP variants were associated with either or both of hearing loss and intellectual disability in at least one individual (58 with intellectual disability and 49 with hearing loss).

To search for patterns of disease severity and extraocular features amongst patients on the Norrie disease/FEVR spectrum associated with each type of variant, we first assessed the consistency of diagnostic classification between authors. We found a high degree of variability in the criteria authors applied in order to determine whether patients with bilateral disease ought to be classified as FEVR or Norrie Disease. We therefore reclassified each reported case as ‘severe’ or ‘moderate’ using fixed criteria based on the retinal phenotype, provided it had been sufficiently described by the authors.

Of 201 unique variants, 92 were reported in papers in which the retinal phenotype was described in sufficient detail to enable reclassification. All but one of the 92 variants (c.362G > A, R121Q) consistently resulted in either ‘severe’ or ‘moderate’ retinal disease. In addition, variants in all but three amino acid residues (regardless of which residue replaced the original amino acid) consistently resulted in either ‘severe’ or ‘moderate’ retinal disease. Prior to reclassification 16 of these 92 variants had been reported as causing both FEVR and Norrie disease in different patients: of these, 14 were upgraded to ‘severe’ retinal disease, one was found to produce a genuinely variable phenotype and one was downgraded to ‘moderate’ retinal disease. Of 124 variants originally reported as causing only Norrie disease, five were downgraded to ‘moderate’ retinopathy. Of 46 variants originally reported as causing only FEVR, 15 were upgraded to ‘severe’ retinopathy. This resulted in 69 variants that consistently caused ‘severe’ disease, 22 that consistently caused ‘moderate’ disease and one that could cause either ‘severe’ or ‘moderate’ disease (Supplementary Table S1).

We found that when FEVR and Norrie disease had been reported in the same family (by the same author) this was due to a difference in the genotype; for example one individual with Norrie disease was hemizygous whereas his relative with FEVR was heterozygous for the same pathogenic NDP mutation. (Li et al., 2018). Reports by different authors of the same variant causing either FEVR or Norrie disease in unrelated patients conflicted primarily due to variability in the authors’ respective case definitions rather than true differences in disease severity between patients.

**FIGURE 2:**
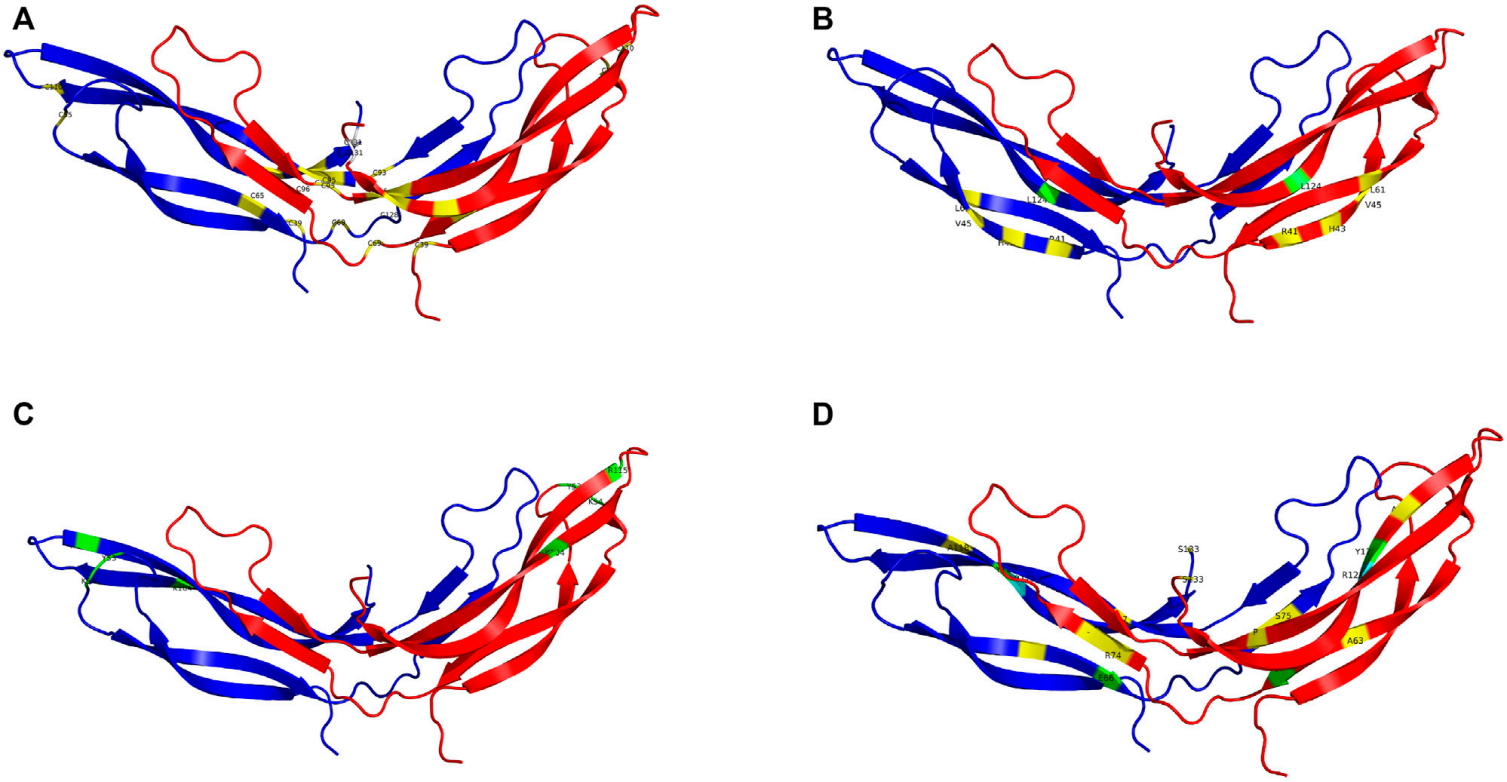
The NDP homodimer is depicted in each panel with one molecule in blue and the other in red. **(A)** shows pathogenic variants in cysteine residues, **(B)** shows pathogenic variants at the FZD4 binding site, **(C)** shows pathogenic variants at the LRP5/6 binding site, **(D)** shows pathogenic variants at the dimer interface. Mutations that cause severe retinopathy are shown in yellow whilst those that cause moderate retinopathy are shown in green. Mutations that result in a retinal phenotype of variable severity are shown in cyan. All molecular scale representations were prepared using the PyMOL Molecular Graphics System (Version 2.6.0a0, Schrödinger, LLC) [Schrodinger, LLC PyMOL (TM) Molecular Graphics System, Version 2.6.0a0]. The structure of norrin protein was from the Protein Data Bank (PDB ID = 5BQB) [Chang, T.-H, et al., Structure and functional properties of Norrin mimic Wnt for signaling with Frizzled4, Lrp5/6, and proteoglycan. eLife, 2015. 4: p. e06554].

Pathogenic variants were classified into groups based on the nature of the mutation as follows: Splice site, UTR, Missense, Nonsense/Frameshift, Large insertions, deletions and inversions. Missense mutations were further sub-categorised by the affected protein region as defined by Ke et al., in 2013 (Ke et al., 2013); cysteine mutations, FZD4 binding site, LRP 5/6 binding site/Dimer interface/Hydrophobic packing/Signal peptide (Figure 2; Supplementary Videos S1–4).

**FIGURE 3:**
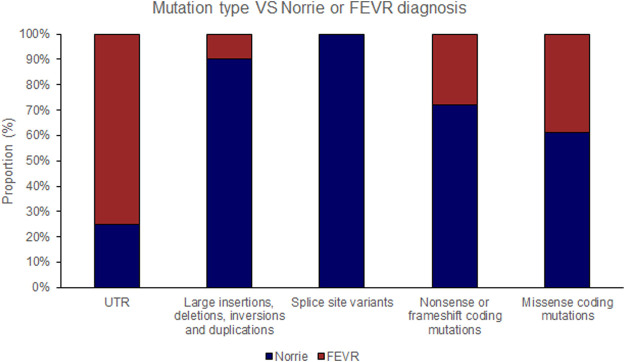
Pathogenic variants (‘mutations’) were classified by type (UTR [untranslated region], large insertions, deletions, inversions and duplications, splice site variants, nonsense or frameshift coding mutations and missense coding mutations). The proportion of mutations of each type that causes either Norrie disease or FEVR, as labelled by the authors of each case report, is shown.

**FIGURE 4:**
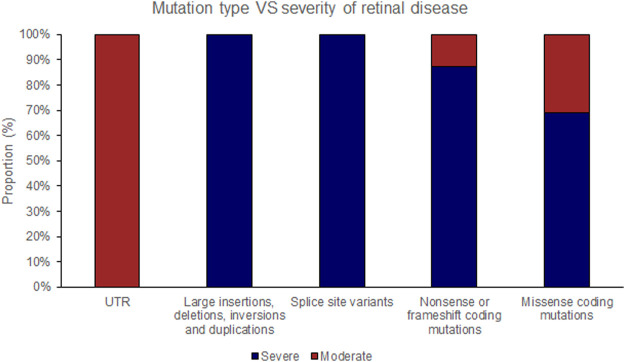
Pathogenic variants (‘mutations’) were classified by type (UTR [untranslated region], large insertions, deletions, inversions and duplications, splice site variants, nonsense or frameshift coding mutations and missense coding mutations). The proportion of mutations of each type that causes either ‘severe’ or ‘moderate’ retinopathy is shown.

The position of the substituted amino acid in the NDP protein predictably determined whether the mutation resulted in ‘severe’ or ‘moderate’ disease with only three exceptions (Table 4; Supplementary Table S1). Large deletions/insertions/inversions/duplications and splice site variants invariably caused ‘severe’ retinopathy whilst UTR mutations only caused moderate retinopathy (Figures 3, 4).

**TABLE 4:** Missense mutations within the NDP coding DNA were classified by the functional region of the NDP protein affected. The table shows the relationship between the region of missense variants and their resulting pathology. Mutations are listed in order of amino acid residue number (aa#). The severity of retinal disease according to the classification system described in the methods is recorded along with the presence or absence of hearing loss and intellectual disability. The diagnoses of FEVR, Norrie disease, or both FEVR and Norrie disease (as stated by the authors of the case reports of mutations in each amino acid residue) are recorded.

		Mutation in Aa #	Amino Acid Change	Retinal Severity	Learning Disability	Hearing Loss	Diagnosis in Original Paper
Cysteine mutations		39	C39R	Severe		Y	Norrie
		55	C55R; C55F; C55Y	Severe			Norrie and FEVR
		65	C65Y; C65W	Severe			Norrie and FEVR
		69	C69R; C69S	Severe	Y	Y	Norrie
		93	C93R	Unclear		Y	Norrie
		95	C95R; C95F; C95W; C95Y	Severe			Norrie and FEVR
		96	C96Y; C96F; C96W	Severe	Y		Norrie
		110	C110G; C110R	Severe		Y	Norrie and FEVR
		126	C126R; C126F; C126S	Severe	Y	Y	Norrie and FEVR
		128	C128R; C128F	Severe			Norrie
		131	C131G	Unclear			FEVR
FZD4 binding site mutations	Beta 1/2 region	41	R41T; R41K; R41S	Severe	Y	Y	Norrie and FEVR
		43	H43N; H43R; H43Q	Severe	Y	Y	Norrie and FEVR
		45	V45M; V45E; V45G	Severe	Y		Norrie and FEVR
		61	L61I; L61F; L61P	Severe		Y	Norrie and FEVR
	Beta 3/4 region	124	L124F	Moderate			FEVR
LRP5/LRP6 binding site mutations	53	Y53C	Moderate			FEVR	
		54	K54N	Moderate			FEVR
		104	K104Q; K104N	Moderate			Norrie and FEVR
		115	R115L	Moderate			FEVR
Dimer interface	44	Y44C	Unclear	Y		Norrie and FEVR	
		62	L62P	Unclear			Norrie
		63	A63S; A63V; A63D	Severe			Norrie and FEVR
		66	E66K	Moderate			FEVR
		67	G67R; G67E; G67V	Unclear			Norrie and FEVR
		74	R74C	Severe			Norrie
		75	S75P; S75C	Severe	Y	Y	Norrie
		89	F89L	Unclear	Y		Norrie
		97	R97P	Severe		Y	Norrie
		98	P98L; P98R	Unclear		Y	Norrie
		118	A118D	Severe			Norrie
		120	Y120C	Moderate			FEVR
		121	R121Q; R121L; R121G	Variable	Y	Y	Norrie and FEVR
		123	I123N	Unclear	Y	Y	Norrie
		133	S133C	Severe	Y		Norrie
Hydrophobic packing	60	V60E	Severe	Y		Norrie	
		90	R90C; R90H; R90P	Unclear	Y	Y	Norrie
		92	S92P	Severe		Y	Norrie
		101	S101F	Unclear			Norrie
		103	L103V; L103Q	Variable			Norrie and FEVR
		105	A105P; A105T; A105E	Variable			Norrie and FEVR
		123	I123N	Unclear	Y	Y	Norrie
Met 1	1	M1V, M1R, M1K, M1T	Severe	Y	Y	Norrie	
Signal peptide	8	A8P	Unclear			FEVR	
		13	L13R	Severe	Y	Y	Norrie
		16	L16P	Severe			Norrie
		18	I18K	Moderate			FEVR

**FIGURE 5:**
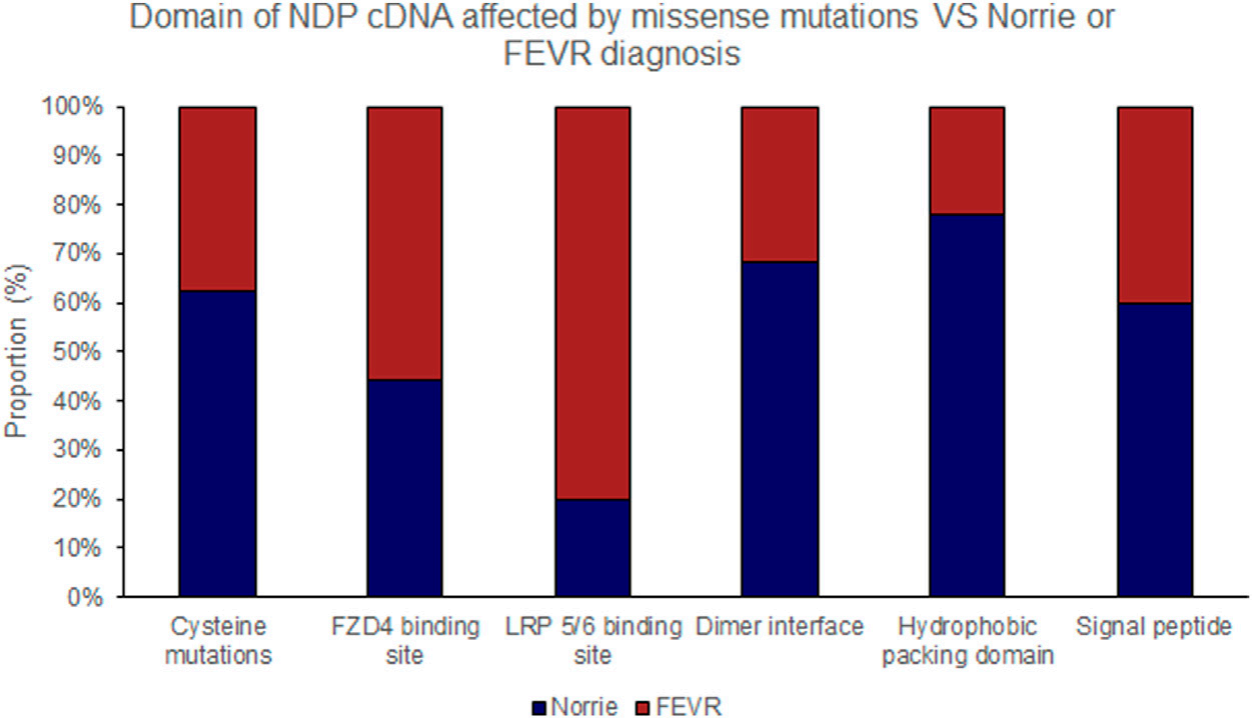
Missense mutations within the NDP coding DNA were classified by the functional region of the NDP protein affected. The proportion of mutations of each type that causes either Norrie disease or FEVR, as labelled by the authors of each case report, is shown.

**FIGURE 6:**
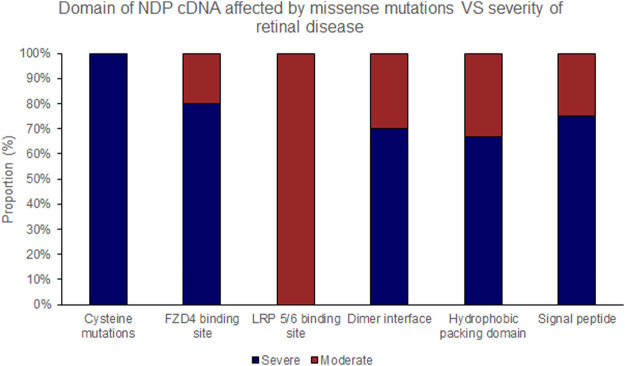
Missense mutations within the NDP coding DNA were classified by the functional region of the NDP protein affected. The proportion of mutations of each type that causes either ‘severe’ or ‘moderate’ retinopathy is shown.

Missense mutations within the cDNA could cause either severe or moderate retinopathy, however when missense mutations were sub categorised by the functional region of the NDP protein affected, cysteine mutations were found to universally result in severe retinopathy whereas LRP 5/6 binding site mutations universally caused moderate disease (Table 4; Figures 2, 5, 6). In silico pathogenicity prediction for missense mutations was performed, generally producing high CADD scores (mean 26.4).

**FIGURE 7:**
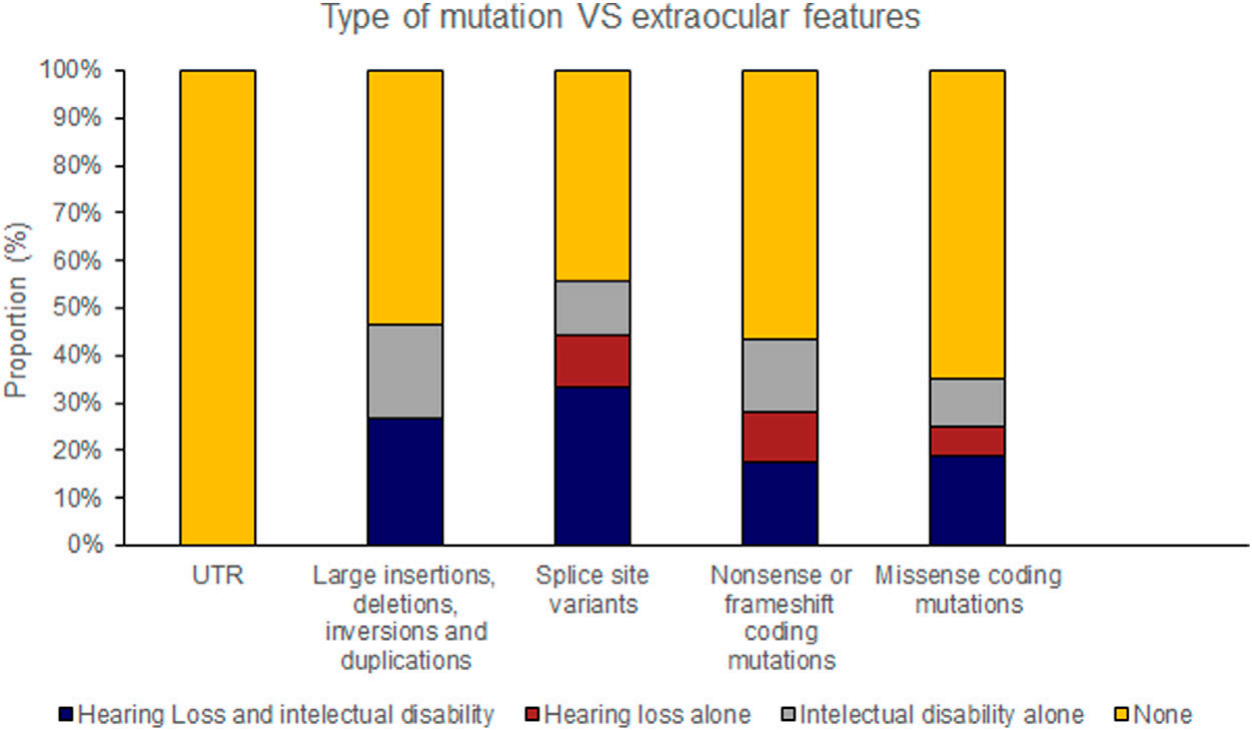
Pathogenic variants (‘mutations’) were classified by type (UTR [untranslated region], large insertions, deletions, inversions and duplications, splice site variants, nonsense or frameshift coding mutations and missense coding mutations). The proportion of mutations of each type that caused extraocular features (hearing loss or intellectual disability) is shown.

**FIGURE 8:**
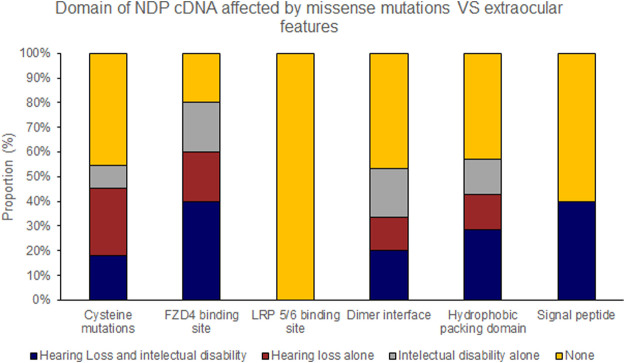
Missense mutations within the NDP coding DNA were classified by the functional region of the NDP protein affected. The proportion of mutations of each type that caused extraocular features (hearing loss or intellectual disability) is shown.

Hearing loss and intellectual disability were almost exclusively associated with ‘severe’ (25 of 70 variants) rather than ‘moderate’ retinal disease (1 of 23 variants) (Table 4; Supplementary Table S1). Extraocular features were most likely to be associated with missense mutations affecting the FZD4 binding region of NDP whereas there were no cases of extraocular features associated with the LRP 5/6 binding site region of NDP or when the mutation was found in the UTR (Figures 7, 8).

The three patients with Coats disease had either a somatic variant or were heterozygous for a variant that is known to cause ‘severe’ retinopathy akin to Norrie Disease when patients are hemizygous or homozygous.

Variants associated with ROP were predominantly found in the 5′ and 3′ UTR regions. Of variants outside the UTR’s, one was intronic, four were in the coding DNA although one of these was a synonymous variant. Two of the variants associated with ROP have also been associated with FEVR.

n addition to the reported ocular-disease causing variants in NDP, 34 commonly occurring variants were identified from the gnomAD database (Table 5). They had high minor allele frequencies, generally low CADD scores (mean 14.0) and are expected to be benign. 26 of these were synonymous variants and were found throughout the gene. None of the synonymous variants were found to affect splicing using the in silico prediction tool ‘Splice AI’. (No variants had a Splice AI score of over 0.8, which would indicate variants that are strongly predicted to disrupt existing splice sites). Eight variants were missense but were found predominantly in the proximal half of the cDNA, outside of the signal peptide.

**TABLE 5:** List of commonly occurring benign variants in NDP according to the gnomAD database. They are either synonymous mutations or missense mutations that are found to be “benign” and “tolerated” based on results of the Polyphen and SIFT pathogenicity predictors respectively. Splice AI and PHRED-like scaled CADD scores are also included.

Commonly Occuring Benign Variants				Splice AI				In Silico Pathogenicity Prediction		
Variant	Protein Consequence	Annotation	Minor Allele Frequency per Million (gnomAD)	Acceptor Loss	Donor Loss	Acceptor Gain	Donor Gain	Polyphen Score	SIFT Score	PHRED-like Scaled CADD Score
c.42C > T	p.Ser14Ser	synonymous variant	5	0	0	0	0	Benign	Tolerated	10.9
c.78G > A	p.Thr26Thr	synonymous variant	5	0	0	0	0	Benign	Tolerated	4.65
c.95T > C	p.Met32Thr	missense variant	5	0	0	0	0	Benign	Tolerated	16.66
c.102G > A	p.Ser34Ser	synonymous variant	22	0	0	0	0	Benign	Tolerated	4.337
c.101C > T	p.Ser34Leu	missense variant	34	0	0	0	0	Benign	Tolerated	22.4
c.110G > A	p.Arg37GLn	missense variant	5	0	0	0	0	Benign	Tolerated	16.89
c.129C > T	p.His43His	synonymous variant	5	0	0.07 (-45bp)	0	0	Benign	Tolerated	9.68
c.145A > C	p.Ser49Arg	missense variant	11	0	0	0	0.07 (-29bp)	Benign	Tolerated	22.6
c.157T > C	p.Tyr53His	missense variant	5	0	0	0	0.02 (-17bp)	Benign	Tolerated	23
c.162G > A	p.Lys54Lys	synonymous variant	5	0	0.06 (-12bp)	0	0	Benign	Tolerated	10.62
c.165T > C	p.Cys55Cys	synonymous variant	234	0	0	0	0.14 (-9bp)	Benign	Tolerated	13.24
c.166A > G	p.Ser56GLy	missense variant	5	0	0	0	0.12 (-8bp)	Benign	Tolerated	22.8
c.171A > T	p.Ser57Ser	synonymous variant	5	0	0.01 (-3bp)	0	0	Benign	Tolerated	12.16
c.183C > G	p.Leu61Leu	synonymous variant	6	0	0	0	0	Benign	Tolerated	15.19
c.186G > T	p.Leu62Leu	synonymous variant	50	0	0	0	0	Benign	Tolerated	18.29
c.184C > T	p.Leu62Leu	synonymous variant	6	0	0	0	0	Benign	Tolerated	17.58
c.190A > C	p.Arg64Arg	synonymous variant	6	0	0	0	0	Benign	Tolerated	18.56
c.195C > T	p.Cys65Cys	synonymous variant	43	0	0	0	0	Benign	Tolerated	4.077
c.204C > T	p.His68His	synonymous variant	24	0	0	0	0	Benign	Tolerated	16.13
c.216G > A	p.Ala72Ala	synonymous variant	16	0	0	0	0	Benign	Tolerated	0.727
c.225C > T	p.Ser75Ser	synonymous variant	29	0.01 (50bp)	0	0	0	Benign	Tolerated	0.701
c.226G > C	p.Glu76GLn	missense variant	6	0	0	0	0	Benign	Tolerated	22.8
c.232T > C	p.Leu78Leu	synonymous variant	6	0	0	0	0	Benign	Tolerated	12.12
c.240G > A	p.Ser80Ser	synonymous variant	6	0	0	0	0	Benign	Tolerated	13.96
c.255C > G	p.Leu85Leu	synonymous variant	12	0	0	0	0	Benign	Tolerated	19.27
c.274T > A	p.Ser92Thr	missense variant	47	0	0	0	0	Benign	Tolerated	20.9
c.291G > A	p.Arg97Arg	synonymous variant	6	0	0	0	0	Benign	Tolerated	13.76
c.307C > T	p.Leu103Leu	synonymous variant	6	0	0	0	0	Benign	Tolerated	17.85
c.319C > A	p.Arg107Arg	synonymous variant	6	0	0	0	0	Benign	Tolerated	8.238
c.348C > T	p.Leu116Leu	synonymous variant	7	0	0	0	0	Benign	Tolerated	17.75
c.354C > A	p.Ala118Ala	synonymous variant	14	0	0	0	0	Benign	Tolerated	16.85
c.372C > T	p.Leu124Leu	synonymous variant	13	0	0	0	0	Benign	Tolerated	15.9
c.384C > T	p.Cys128Cys	synonymous variant	31	0	0	0	0	Benign	Tolerated	1.122
c.387G > A	p.Glu129GLu	synonymous variant	149	0	0	0	0	Benign	Tolerated	14.82

## 4 Discussion

This review identified 201 different variants in the NDP gene that have been reported as ocular disease-causing. The pathological phenotype that may result from a disease-causing NDP variant is quite diverse but generally comprises a consistent cluster of features that vary predictably with severity of retinopathy. Mutations with the mildest predicted impact on protein function are likely only to produce retinopathy in the context of prematurity. Mutations with a more significant impact will produce ‘moderate’ retinopathy akin to FEVR and mutations with the most severe impact are likely to produce ‘severe’ retinopathy akin to Norrie disease. Previous reviews have found no clear pattern in the nature of NDP mutations and their association with either FEVR or Norrie disease (Nikopoulos et al., 2010), with the exceptions that cysteine residue mutations have been associated with Norrie Disease (Wu et al., 2007) and that truncating mutations are associated with more severe visual loss than missense mutations (Smith et al., 2012). Here we demonstrate that the severity of the retinopathy associated with each mutation is in fact very predictable and depends on the protein region affected.

A key limitation of previous reviews has been variability in the case definition of Norrie disease and FEVR amongst different authors. For example, patients with bilateral total retinal detachments from infancy have occasionally been diagnosed with FEVR as opposed to Norrie disease due to the absence of hearing loss or intellectual disability (Li et al., 2018). In one example a patient with bilateral total retinal detachments from a young age and the following mutation [c.134T > Gm p. V45G] was diagnosed with FEVR on the basis that stage 1 FEVR was found in his mother (who was heterozygous for the mutation) and that he did not have any hearing loss or intellectual disability (Li et al., 2018). We believe this is not justified because firstly, the diagnosis of Norrie Disease does not require patients to exhibit these extraocular features and secondly, if hearing loss is to occur it often begins to manifest in the second or third decade of life whereas the cases reported on often relate to children in their first decade. Conversely, sometimes patients have been diagnosed with Norrie disease with isolated vitreoretinal disease that is less severe than most other children with Norrie disease and could easily be considered FEVR by a different clinician. For example, Meindl et al. described four children with NDP mutations who were diagnosed with Norrie disease after presenting with unilateral or symmetric retinal folds and vitreous haemorrhage rather than retrolental masses. They did not progress to bilateral total retinal detachment and did not develop hearing loss or intellectual disability (Meindl et al., 1995).

Since the severity of retinal disease in the reported cases of FEVR and Norrie disease considered in this review form a continuum rather than a bimodal distribution, and since the mechanism of disease is fundamentally the same (NDP mutation) in each case, we propose it is justifiable to combine Norrie disease and FEVR into a single group and then stratify patients based on the severity of vitreoretinal disease described in each case report. The advantage of using this approach to classify patients as “severe” or “moderate” rather than the more traditional diagnostic labels of “FEVR” and “Norrie Disease” that pre-date genetic medicine is that it is possible to achieve far greater consistency between case reports. This in turn may better predict associated non ocular features.

We thus classified patients into two groups based only on the severity of their retinal disease as described in the methods section. Following reclassification we conclude that the majority of mutations are responsible for a consistent phenotype in each case. Furthermore, the severity of the retinal phenotype resulting from mutations within the coding DNA can be predicted based only on which of the 133 amino acids in the NDP protein had been affected. There are three notable exceptions to our conclusion; missense mutations affecting amino acids 103, 105 and 121, which could cause either ‘severe’ or ‘moderate’ disease even when the amino acid change was identical. This may be because they cause disease severity on the threshold between ‘moderate’ and ‘severe’ and therefore the phenotype is likely to depend heavily on the timing of presentation.

It should however be noted that this conclusion is based upon incomplete information as only 90 out of 219 papers reporting pathogenic variants described the phenotype in sufficient detail to enable classification.

Having established that most pathogenic variants result in a consistent phenotype, we investigated whether the phenotype could be explained by the nature and/or location of the protein change (at the molecular level) induced by each variant. Using a methodology similar to that employed by Juyuan Ke et al., we separately assessed the effect of large deletions, frame shift/early termination of translation, coding mutations (by functional region) and untranslated region mutations with respect to the resulting phenotype (severity of retinal disease in patients with Norrie/FEVR, the development of hearing loss or intellectual disability, Coats disease, retinopathy of prematurity and other rarer phenotypes) (Ke et al., 2013).

The trends found are discussed in the following sections and set into the context of other relevant literature:

### 4.1 Variants Associated With Severe/Moderate Retinal Disease Along the Norrie/FEVR Spectrum

#### 4.1.1 Large Deletions Cause Severe Retinal Disease

**FIGURE 9:**
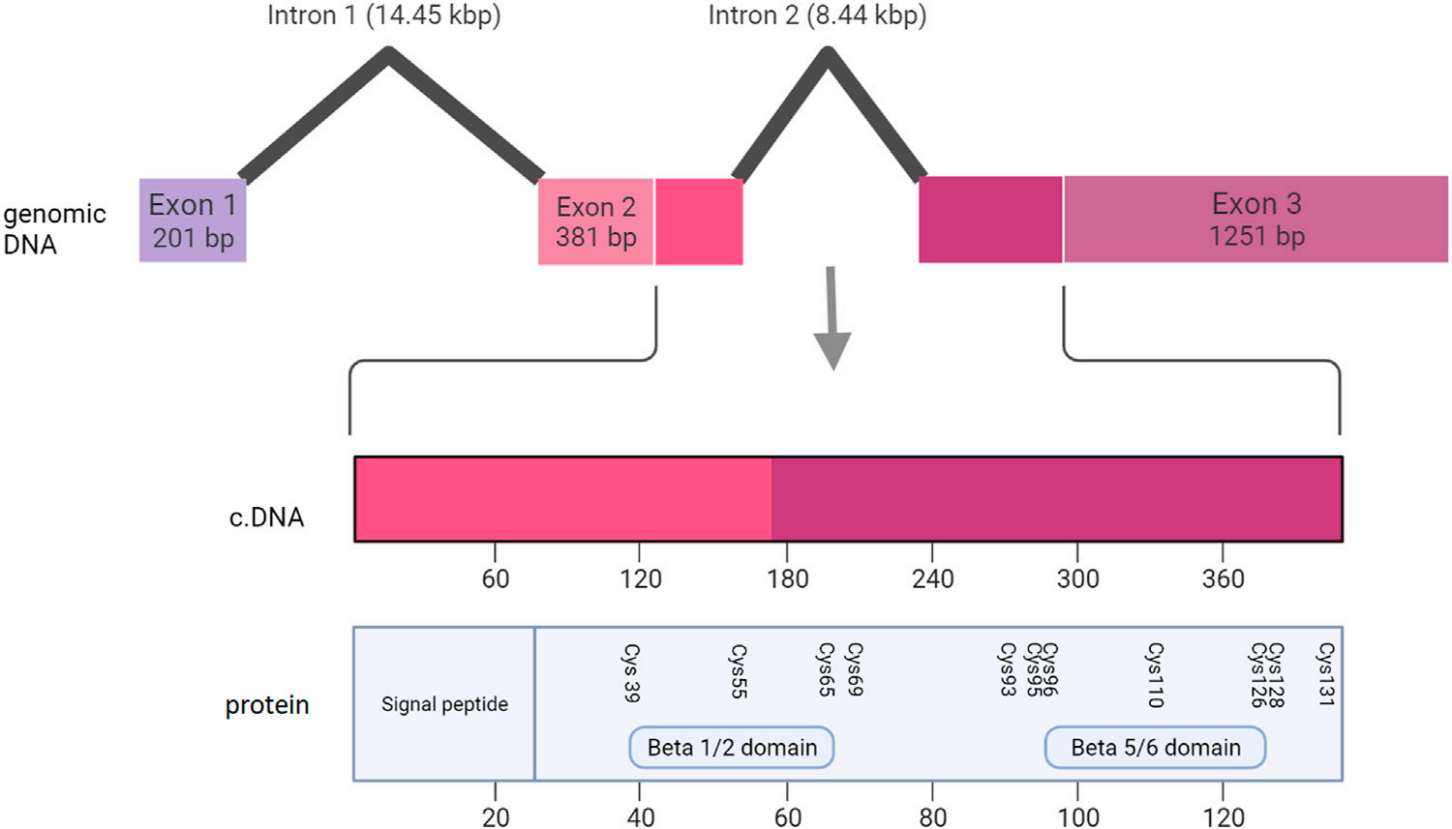
Schematic representation of the NDP gene, showing the relationship between the genomic DNA, the coding DNA (cDNA) and the functional regions of the NDP protein.

Large deletions containing any one of the three NDP exons (Figure 9) were universally associated with bilateral total retinal detachments. This was true of all 5 cases described in sufficient clinical detail to establish the retinal status. In addition, all of the other 16 cases of large insertions and deletions that were described in less clinical detail were diagnosed with Norrie Disease as opposed to FEVR. Many of these patients also went on to develop other features of Norrie disease including intellectual disability and hearing loss. The coding sequence of NDP is split across part of exon two and exon 3. Exons two and three encode FZD4 binding sites (beta one to two loop and beta five to six loop respectively) (Chang et al., 2015). The norrin dimer interface is encoded by exon 3. Both of these are predicted to be necessary for successful activation of the norrin/beta catenin pathway (Chang et al., 2015). It is therefore unsurprising that loss of either exon two or three universally results in severe disease. The finding that exon one loss alone can also cause severe disease highlights the equal importance of the 5’ untranslated region (UTR) in the regulation of NDP translation.

#### 4.1.2 Frame Shifts and Early Termination of Translation Cause Severe Retinal Disease

Frame shift mutations and missense mutations that resulted in early termination of translation almost universally cause severe retinal disease (24 of 27 cases). Three exceptions to this rule have been reported (c.69delC, p. D23E fs*9, c.285C > A, p. C95*, and c.378T > A, p. C126*). In all three cases the severity of the retinal disease was only just under the threshold for labelling them as ‘severe’ according to our classification system described above.

Our findings in relation to frame shift, early termination of translation and large deletions are consistent with findings by Smith et al., 2012 (Smith et al., 2012). Although their study is restricted to patients who had been diagnosed with Norrie disease, they found that light perception was significantly more likely to be retained beyond 1 year of age in patients with non-truncating variants as compared to those with truncating variants. Hence, just as in this review, they demonstrate that when the severity of disease is judged based on the examination findings rather than the diagnostic label attributed to each child, a clear genotype-phenotype pattern begins to emerge.

Missense mutations in the coding DNA cause moderate retinal disease when at the LRP5/6 binding sites and severe retinal disease when at the FZD4 binding sites or when a cysteine is lost.

Missense mutations in the coding DNA may cause either ‘severe’ or ‘moderate’ disease. We examined each functional region of the NDP protein separately to determine whether the severity of retinal disease could be predicted based on the region affected.

#### 4.1.3 Cysteine Mutations

Cysteine mutations universally cause “severe” retinal disease (9 of 9 cases). Missense mutations affecting cysteine residues are predicted to have a critical impact on the structure of the norrin homodimer as these residues are required for the formation of cysteine intermolecular disulphide bridges. This finding concurs with the patterns previously recognised and reported by other authors (Wu et al., 2007).

#### 4.1.4 FZD4 and LRP5/6 Binding Site Mutations

Norrin contains separate binding sites for its receptor, FZD4, and for the co-receptors LRP5 and LRP6. Variants in norrin can be grouped into two classes depending on which interaction they disrupt. There is evidence from systematic mutagenesis experiments that two such classes do indeed exist; mutations involving R41, H43, Y44, V45, M59, V60, L61, Y120, Y122 and L124 affect the binding of norrin to FZD4 whereas mutations involving L52, Y53, K54, K104, R107, R109, R115 affect the binding of norrin to its LRP5/6 co-receptors (Ke et al., 2013; Shen et al., 2015). Furthermore, norrin dependent Topflash reporter assays have demonstrated reduced signalling activity when either the FZD4 binding site or the LRP5 binding site is mutated, however if there are mutations simultaneously at both sites, a synergistic effect occurs and signalling activity is much further reduced (Qin et al., 2008). Further evidence of the important contributions of both FZD4 and LRP5 to the signalling pathway comes from animal knockout models demonstrating that absence of either LRP5 (but not LRP6) or FZD4 results in impaired retinal vascular development (Qin et al., 2008; Paes et al., 2011; Chen et al., 2012).

Our results show that there is a clear association between mutations in the FZD4 binding site and ‘severe’ retinal disease (severe in four out of 5 cases) whereas LRP5/6 binding site mutations universally caused moderate disease (moderate in four out of 4 cases). All mutations in the beta 1/2 regions of the FZD binding site caused severe disease whereas mutations in the beta 5/6 FZD binding site resulted in moderate disease. We defined beta 1/2 and 5/6 regions as per the work of Chang et al., 2015 (Chang et al., 2015).

Interestingly, one such mutation in the beta 1/2 region (c.134 T > G, p. V45G) that was reported to cause severe retinal disease akin to Norrie disease was also found to cause FEVR in a heterozygous female, raising the possibility of haploinsufficiency in some cases. A potential explanation as to why haploinsufficiency may manifest in some patients but not others is because of a greater or lesser degree of skewed X inactivation.

Our analysis found that all mutations in the beta one to two regions of the FZD4 binding site (9 reported) caused severe disease whereas all mutation in the beta five to six region or in the LRP 5/6 binding site (6 reported) caused moderate disease.

Previous studies investigating the association between the functional region of norrin affected by missense mutations and the resulting ocular disease (Norrie or FEVR) have not found any clear relationships (Ke et al., 2013). We believe the apparent disagreement between our review and previous work can be resolved by reclassifying the diagnosis (FEVR or Norrie Disease) in each case as ‘severe’ or ‘moderate’ retinal disease according to the criteria that we have outlined above. For example, Jiyuan Ke et al. categorise NDP mutations by the functional region affected [(Ke J, 2013), Supplementary Table S2] and show a mixture of diagnoses caused by cysteine mutations, FZD4 binding site mutations and LRP5/6 binding site mutations. However, if their cases are classified into ‘severe’ and ‘moderate’ groups as according to our system, all of the LRP binding sites result in FEVR-like ‘moderate’ retinopathy, all but one of their listed FZD4 binding sites produce a norrie-like ‘severe’ phenotype and all of their listed cysteine mutations result in a norrie-like ‘severe’ phenotype.

Our findings imply that the interaction between norrin and FZD4 is more important to the integrity of the norrin signalling pathway than the norrin/LRP interaction. Therefore defective FZD4 function ought to produce ‘severe’ retinal disease whereas defective LRP5 function ought to produce ‘moderate’ disease. A topflash reporter assay has demonstrated that a nonsense mutation in FZD4 completely abolishes its signalling activity (Fuchs et al., 1994). There are numerous reports of autosomal dominant FEVR (‘moderate’ disease) that arise either as a result of a heterozygous FZD4 or heterozygous LRP5 mutation. However, a homozygous mutation in FZD that significantly affects protein function ought to produce severe disease whereas a similarly severe homozygous mutation in LRP5 ought to produce ‘moderate’ disease.

Khan et al. (2017) reported on a case of two brothers with a homozygous deletion in FZD4 resulting in a frame shift and early termination of translation (Khan et al., 2017). Although described as “FEVR”, their phenotype would fall into our ‘severe’ category. Both had bilateral total retinal detachments in infancy, with the older sibling having already progressed to bilateral phthisis by the time the younger presented and the paper was written. As they are both still in their first decade of life, it is too early to determine whether hearing loss will develop and therefore at present their phenotype is, in our view, entirely indistinguishable from severe Norrie Disease.

There is increasing awareness that the clinical phenotype known as Norrie disease may not be exclusive to mutations in the NDP gene (Royer et al., 2003) and there is a strong case for recessively inherited FZD4 mutations as a candidate gene in unsolved cases (Khan et al., 2017). Conversely, homozygous mutations in LRP5 tend to produce ‘moderate’ disease (Jiao et al., 2004).

#### 4.1.5 N-Terminal Signalling Peptide

Just five pathogenic missense variants have been found to affect the N-terminal part of norrin. However, three of them result in a ‘severe’ retinal phenotype. Methionine one missense mutations are expected to cause a significant decrease in protein function due to a failure to initiate translation. However, the severity of the phenotype caused by mutations in residues 13 and 16 is consistent with previous work suggesting that the N-terminal signal peptide (amino acids 1–24) performs a critical function: It is thought to be important for transport of the protein from cytosol to endoplasmic reticulum and is a characteristic feature of proteins that are secreted (Von Heijne, 1986; Berger et al., 1992; Fuchs et al., 1994). Despite the critical function of the signal peptide, pathogenic variants are relatively sparse in this region, suggesting that the identity of only a handful residues are critical for its function whilst other mutations are unlikely to affect protein function. Indeed, known non-pathogenic missense single nucleotide polymorphisms that result in amino acid substitutions are clustered in the N′ terminal part of the protein.

#### 4.1.6 Dimer Interface

Mutations affecting the dimer interface, including the c-terminal serine, tended to cause severe disease. This was seen in five out of six variants with an adequately described retinal phenotype, although c.196G > A, p. E66K is a clear exception to this rule. This is consistent with previous work that has shown norrin to function as a homodimer (Chang et al., 2015).

#### 4.1.7 Hydrophobic Packing Residues

Mutations in the hydrophobic packing residues could cause either ‘severe’ or ‘moderate’ retinal disease and both Norrie disease and FEVR has been reported, although ‘severe’ disease has been reported more often than ‘moderate’ (4 variants vs. Two variants). Mutations in the hydrophobic packing region are likely to lead to a change in the tertiary structure of the protein. Interestingly a mutation in residues L103 or A105 could result in either ‘severe’ or ‘moderate’ disease depending on the amino acid that was substituted in.

#### 4.1.8 Untranslated Region and Splice Site Mutations

Several variants in the splice sites and UTR’s have been attributed to Norrie Disease and FEVR. Following reclassification of the severity of retinal disease as ‘severe’ or ‘moderate’, the phenotype associated with these mutations was found to vary widely. Analysis of these variants was impeded by a paucity of papers with detailed descriptions of the retinal clinical phenotype (only two of 13 papers).

### 4.2 Variants Associated With Intellectual Disability and Hearing Loss

There is a clear association between a ‘severe’ retinal phenotype, hearing loss and intellectual disability. These extraocular features were only found in patients with ‘severe’ retinopathy although not all patients with the ‘severe’ retinal phenotype were reported to develop these extraocular features even when the proband was into their fourth decade of life. When the coding region of NDP was examined, variants that resulted in a frame shift and premature termination of translation were commonly associated with hearing loss and intellectual disability. Missense mutations in the cysteine residues, the dimer interface or the hydrophobic regions were associated with intellectual disability or hearing loss in half of cases. Missense mutations in the beta one and two regions of the FZD4 binding site were associated with either intellectual disability or hearing loss in two thirds of cases, and every case of ‘severe’ retinal disease caused by a missense mutation in the beta one or beta two regions of the FZD4 binding site was associated with either hearing loss or intellectual disability. There were no cases of either hearing loss or intellectual disability when a pathogenic variant occurred in the beta 5/6 FZD4 binding region or in any case of an LRP 5/6 binding site mutation. Mutations in Methionine one were commonly associated with severe retinal disease, hearing loss and intellectual disability. Of the other four variants reported in the N-terminal signal peptide, only one was associated with intellectual disability and hearing loss.

There are two parallel hypotheses relating to the disease mechanism of intellectual disability in Norrie Disease when caused by a large deletion: The first is that large deletions of the NDP gene are often accompanied by deletions of the monoamine oxidase (MAO) genes. In such cases, the putative mechanism is a dysfunction of catecholamine metabolism as the cause of the intellectual disability and the NDP deletion as the cause of the ocular phenotype. There is good evidence that a deletion of MAO A alone can cause intellectual disability (Brunner et al., 1993). The second is that intellectual disability arises when norrin signalling is severely disrupted by a large deletion in NDP, resulting in maldevelopment of the cerebral vasculature. Interestingly all cases of NDP deletion that coincide with a MAO A or B deletion, intellectual disability is seen together with a severe retinal phenotype but without hearing loss. However, when the NDP deletion involves exons 1,2 and 3 (alone or in combination with each other) but is not associated with a contiguous Monoamine Oxidase A or B deletion, intellectual disability is present in just half of cases and is usually associated with hearing loss. Taken together, these findings suggest that MAO A and B deletions cause intellectual disability independent of the NDP deletion, however if an NDP deletion causes a severe enough loss of function it may alone cause intellectual disability.

### 4.3 Variants Associated With Retinopathy of Prematurity

NDP variants associated with retinopathy of prematurity are predominantly found in the 5′ and 3′ untranslated regions. There is one report of ROP in a male patient with premature termination of translation (c.131dupA, p. Y44X (Hatsukawa et al., 2002)) and two reports of ROP in patients with missense mutations (p.L108P in a male and p. R121W in heterozygous females (Shastry et al., 1997)). There was also a report of a synonymous variant enriched in advanced ROP (c.186 G- > T) (Haider et al., 2002). As there are likely to be many non-pathogenic polymorphisms in the untranslated region of NDP and even severe mutations in the coding region of NDP must usually be homozygous before causing disease, it is difficult to conclude with certainty that the variants found contribute to the retinopathy of prematurity diagnosed in these cases. The c.186 G- > T synonymous variant is very unlikely to cause incorrect splicing based on ‘Splice AI’ in-silico prediction and had a reasonably high minor allele frequency of 50 per million (as per the gnomAD database). A large genetic study of premature neonates with and without retinopathy of prematurity would be required to determine whether these variants are enriched in those that go on to develop ROP; SNPs found in healthy adults who were born at term cannot be assumed to be non-pathogenic in pre-term infants. At present there is no such large study of the genetics of retinopathy of prematurity (Swan et al., 2018).

It is however credible that ROP may predominantly arise from UTR mutations when the disease mechanism of ROP is considered: Norrin levels fall in response to hyperoxia (Tokunaga et al., 2013). Therefore if a UTR mutation reduces translation of NDP, the norrin concentration may still be sufficient to support normal vascular BRB development in probands born at term, but in pre-term infants it may become insufficient. Therefore variability in the UTR of NDP may determine the degree of prematurity that different neonates can tolerate before ROP develops. It is perhaps surprising that ROP is not more often associated with variants in the cDNA, although it may be the case that signalling deficiency caused by a structural change in the protein, as opposed to reduced translation, would often be severe enough to cause FEVR or Norrie disease regardless of prematurity.

### 4.4 Variants Associated With Coats Disease

Two variants that are associated with severe retinal disease in hemizygous males (c.47 T > C, p. L16P and c.288 C > G, p.96W) were also associated with Coats disease in heterozygous females within the same family. However, it should be noted that not all heterozygous females within these families developed Coats disease. We therefore concluded that heterozygous females carrying these Norrie-disease variants were at increased risk of Coats disease. This finding is consistent with the theory that Coats disease may arise from a unilateral disease-causing somatic mutation in NDP, which causes Coats disease in males regardless of their genetic background and in females who already carry one disease-causing allele for the condition and then acquire a somatic mutation in the other. Consistent with this theory, a study of the genetic cause of Coats disease in nine enucleated eyes from males with the condition found one of the c.288 C > G, p.96W variant present as a somatic mutation exclusively within the retinal tissue (Black et al., 1999).

### 4.5 NDP Variants Associated With Other Phenotypes

There was one reported case of bilateral persistent foetal vasculature associated with a c.123G > C, p. R41S mutation. However, according to the authors the case was in practice difficult to distinguish from bilateral ND or FEVR. The patient also had co-existing glucose-6-phosphate dehydrogenase deficiency, which may have altered the phenotype (Wu et al., 2007). Interestingly other mutations of arginine 41 (R41T and R41K) cause Norrie disease with a ‘severe’ retinal phenotype as per our classification system. R41T has additionally been associated with hearing loss and intellectual disability.

Two cases of a c.293 C > T, p. P98L variant in siblings with microphthalmia and sclerocornea have been reported. The same mutation has previously been reported as a cause of Norrie Disease. The mutation was detected as a result of whole exome sequencing in patients with ocular developmental disorders, therefore we should remain alert to the possibility that this was an incidental finding rather than the causative mutation (Deml et al., 2016).

## 5 Conclusion

A large number of pathogenic variants in NDP have now been reported, causing several related disorders of retinal vascular development.

Each mutation tended to cause quite a predictable phenotype every time it was detected in a patient. This finding is important for determining the prognosis, especially if the diagnosis is made pre-natally or in a carrier female of child bearing age. The potential link between Coats disease, Norrie disease and FEVR is also of importance. In addition hearing loss and intellectual disability developed more often in cases with a ‘severe’ rather than ‘moderate’ retinal phenotype.

Based on our review of reported cases with identified mutations, we conclude that the severity of the phenotype related to NDP mutations is dependent upon the functional region of norrin that is affected. For example, mutations affecting the LRP binding site are likely to cause a milder retinal phenotype than mutations affecting the FZD4 binding site or cysteine residues, which are involved in intermolecular dimerisation. In addition, mutations in the UTR resulting in a failure of translation or frame shift mutations resulting in early termination of translation and non-production of a functional norrin protein are more likely to cause ‘severe’ rather than ‘moderate’ retinopathy.

An understanding of the phenotype that results from the spectrum of NDP mutations will be essential if NDP mutations become treatable with gene therapy in the future. NDP is a good candidate for gene therapy as the expression cassette is short enough to fit into a single AAV genome and because its spatial distribution within the retinal tissue is not thought to be important, therefore it is likely to be deliverable by means of a single intravitreal injection (Ohlmann et al., 2005; Tokunaga et al., 2013).
